# Copper-Promoted One-Pot Approach: Synthesis of Benzimidazoles

**DOI:** 10.3390/molecules25081788

**Published:** 2020-04-14

**Authors:** S. N. Murthy Boddapati, Ramana Tamminana, Ravi Kumar Gollapudi, Sharmila Nurbasha, Mohamed E. Assal, Osamah Alduhaish, Mohammed Rafiq H. Siddiqui, Hari Babu Bollikolla, Syed Farooq Adil

**Affiliations:** 1 Department of Chemistry, Acharya Nagarjuna University, Guntur, Andhar Pradesh 522510, India; snmurthyboddapati@gmail.com (S.N.M.B.); ravig333@gmail.com (R.K.G.); nurbasha@gmail.com (S.N.); 2 Department of Chemistry, Sir C. R. Reddy College, PG Courses, Eluru, Andhra Pradesh 534002, India; 3 Department of Chemistry, GITAM Deemed to Be University, Bengaluru Campus, Karnataka 562163, India; rtamminana17@gmail.com; 4 Department of Chemistry, College of Science, King Saud University, P.O. 2455, Riyadh 11451, Saudi Arabia; masl@ksu.edu.sa (M.E.A.); rafiqs@ksu.edu.sa (M.R.H.S.)

**Keywords:** copper catalyst, desulfurization, domino C–Ncross-coupling, 2-aminoaryl benzimidazole, homogeneous catalysis

## Abstract

A facile, one-pot, and proficient method was developed for the production of various 2-arylaminobenzimidazoles. This methodology is based for the first time on a copper catalyst promoted domino C–N cross-coupling reaction for the generation of 2-arylaminobenzimidazoles. Mechanistic investigations revealed that the synthetic pathway involves a copper-based desulphurization/nucleophilic substitution and a subsequent domino intra and intermolecular C–N cross-coupling reactions. Some of the issues typically encountered during the synthesis of 2-arylaminobezimidazoles, including the use of expensive catalytic systems and the low reactivity of bromo precursors, were addressed using this newly developed copper-catalyzed method. The reaction procedure is simple, generally with excellent substrate tolerance, and provides good to high yields of the desired products.

## 1. Introduction

**Figure 1:**
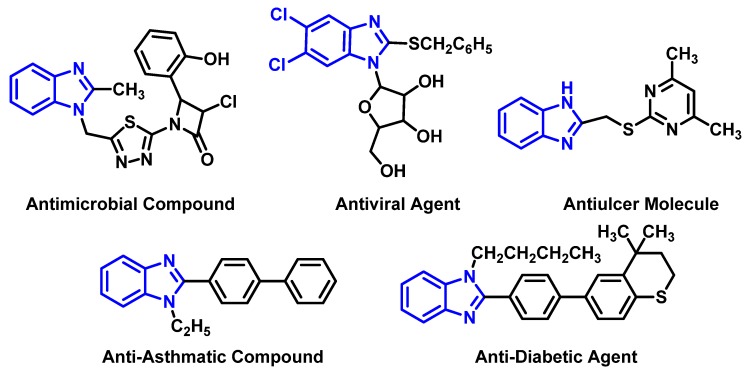
Examples of some biologically active benzimidazoles.

Heterocyclic compounds like benzimidazoles are very important molecules that can be found in a number of natural and synthetically prepared biologically active compounds such as 3-chloro-1-5-(2-methyl-1H-bezimidazol-2-yl)-4-(substituted) phenylazetidin-2-one (an antimicrobial compound [1]), 5, 6-dichloro-1-(β-d-ribofuranosyl)benzimidazoles(an antiviral agent [2]), pyrimidyl-thio-methyl-benzimidazole(an antiulcer molecule [3]), 1-ethyl-2-(4-phenyl)phenyl-1H-benzo[d]imidazole(an antiasthmatic compound [4]), and 1-butyl-2-(3,4-dihydro-4,4-dimethyl-2H-thiochromyl)phenyl-1H-benzo[d]imidazole (an anti-diabetic molecule [4]), shown in Figure 1. Furthermore, these compounds have been identified in N-methyl-d-aspartate (NMDA) antagonists [5], in analgesic [6], anti-inflammatory [7], anti-cancer [8] drugs, in factor Xa(FXa) inhibitors [9], in poly(ADP-ribose)polymerase (PARP) inhibitors [10] and in non-peptide thrombin inhibitordrugs [11].

Thus, enormous efforts have been focused on the preparation of benzimidazole structural frameworks. Classical methods may involve (i) the inter cyclocondensation of 2-aminophenylaniline precursors with either carboxylic acids or aldehydes followed by oxidation [12], (ii) the diazotization on 1-benzimidazolylidenehydrazine [13], (iii) the cyclocondensation of esters with diaminobenzene under microwave irradiation [14], (iv) the reductive cyclization of *o*-nitroaniline with aldehyde using sodium dithionite [15] and (v) a solid-phase route [16]. However, most of these protocols suffer from major shortcomings such as limited suitable substituents for diverse synthesis, strong alkaline conditions, troublesome management of the chemical process, and elevated temperature. Therefore, we reasoned that a catalytic approach involving C–N bond formation would overcome the abovementioned disadvantages.

In the past decade, efficient methods have been described for the synthesis of heterocyclic compounds by aryl halides with copper catalysts [17,18]. Buchwald and co-authors developed the preparation of indulines [19], 2-aryl-4-quinolones [20] and N-alkylbenzimidazoles [21]. The group of Ma described a cascade approach for the production of benzofurans [22], dihydrobenzimidazole-2-ones [23], benzimidazoles [24], isoquinolines [25], pyrrolo [1,2-a]quinoxaline [26], and indoles [27]. Intramolecular C–X bond formation has been established by Batey’s group to synthesize benzoxazoles [28], benzothiazoles [29] and aminobenzimidazoles [30]. In addition, C–N bond formation has been well explored using various transition metals such as copper [31,32], palladium [33], cobalt [34], zinc [35], ruthenium [36] for the production of various heterocycles, but, to the best of our knowledge, no report is available on the synthesis of substituted 2-aminophenylbenzimidazoles employing a copper catalyst through the reporting strategy.

Very recently, 2-phenylaminobenzimidazoles have been reported from thiourea using cobalt catalysis [37]; however, they have failed to develop from bromo precursors. Therefore, in continuation of our research towards the development of efficient methodologies for the synthesis of heterocycles [38,39], herein, we developed a methodology for the synthesis of 2-phenylamino benzimidazoles from molecules having a bromo moiety, under moderate reaction conditions using copper catalysis. Another advantage of our method is the use of copper, which is cheaper, more air-stable and more easily available than cobalt.

## 2. Results and Discussion

**Scheme 1:**
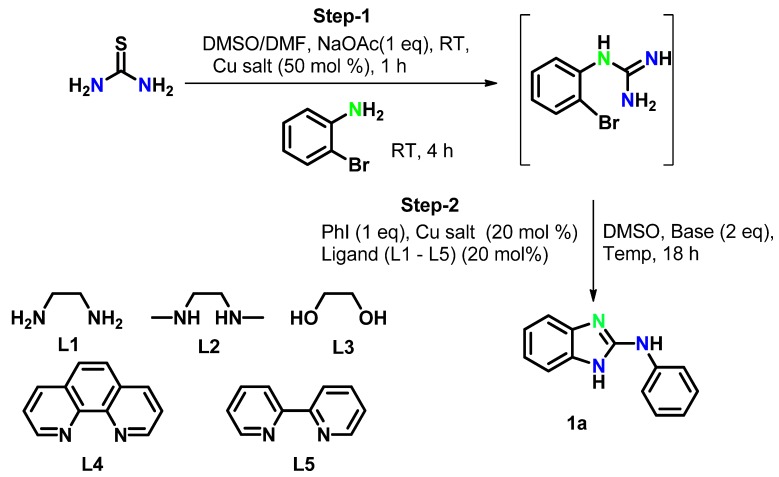
Synthetic route for the synthesis of *N*-phenyl-1*H*-benzo[*d*]imidazol-2-amine.

In this context, 2-phenylaminobenzimidazoles were prepared from thiourea through an approach consisting of consecutive desulphurization/nucleophilic substitution and C–N cross-coupling. Thiourea reacted with 2-bromoaniline at room temperature using copper salt as a catalyst to give N-(2-bromophenyl)-guanidine, which further underwent a domino intra and intermolecular C–N cross-coupling reaction with aryliodide to yield the desired product 2-phenylaminobenzimidazole **1a** (Scheme 1). Optimization was achieved by taking thiourea and bromoaniline as model substrates and using various solvents, bases, ligands, and copper salts at different temperatures. We were pleased to observe that thiourea gave 2-bromoguanidine in quantitative conversion using NaOAc as a base and both copper (I) and copper (II) salts in the presence of DMSO/DMF at room temperature. The reaction was further continued at room temperature using K_2_CO_3_ (2 equiv) as a base, Cu salt (20 mol%), ligand L4 (20 mol%) for 18 h. Unfortunately, no target product was observed (Table 1, entry 1). Later, the reaction was performed at 50 °C, however, no product formed (Table 1, entry 2). In contrast, target product **1a** was observed in 15% yield when the reaction was carried out at 80 °C (Table 1, entry 3). The yield of the product increased to 52% when the reaction was conducted at 100 °C (Table 1, entry 4). Very interestingly, the reaction provided the desired product **1a** quantitatively at 120 °C (Table 1, entry 5).

Bases like K_2_CO_3_ and Cs_2_CO_3_ were found to be effective for the reaction (Table 1, entries 5 and 7), whereas KOH was less effective (Table 1, entry 6). All the tested copper salts, i.e., CuI, CuBr, CuCl, CuSO_4_·5H_2_O, Cu(OAc)_2_·H_2_O, exhibited similar catalytic activity (Table 1, entries 5 and 8–11). Among the tested ligands, L4 was efficient (Table 1, entry 5), while the other ligands L1–L3 and L5 provided the target product in lower yields (Table 1, entries 13–16). In contrast, the reaction didnot proceed in the absence of ligand (Table 1, entry 12).

**Scheme 2:**
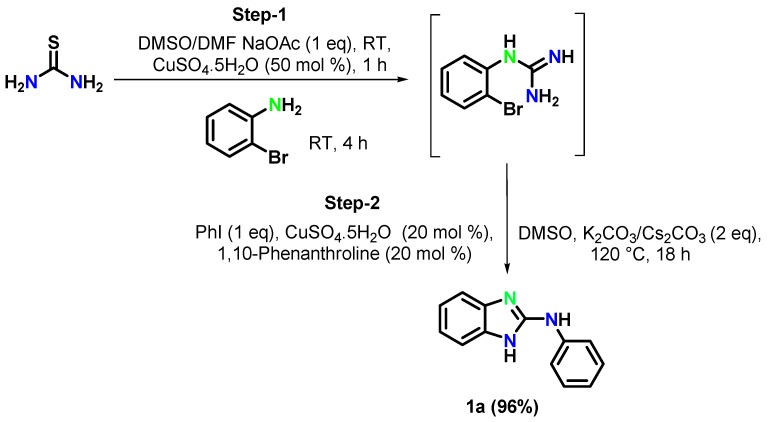
Optimized synthetic pathway for the synthesis of 2-phenylaminobenzimidazole from thiourea.

By lowering the quantity of the copper source (10 mol%) or of the base (1.0 equiv), the reaction led to *N*-arylation, affording a lower amount of the target product (Table 1, entries 17–18). Control experiments also confirmed that the reaction could not yield the target product in the absence of a catalyst, and the intermediate 2-bromophenylguanidine was recovered intact (Table 1, entry 19). This control experiments demonstrated that the reaction requires a catalyst. Despite the similar catalytic activity of all the copper salts tested, considering the cost effectiveness of CuSO_4_·5H_2_O over the other examined catalysts, the authors chosen CuSO_4_·5H_2_O as a reasonable copper source for both step 1 and step 2 in Scheme 1. Finally, the total outcome of the above optimization studies is presented in Scheme 2.

The compound **1a** was further confirmed based on spectral data, for example, the appearance of characteristic peaks at 1542(m), 1478(s), 1413(s) cm^−1^, clearlyshowing Ar–C=C stretching, a peak at 3098(w) cm^−1^ showing aromatic =CH stretching, and peak at 3294(s) cm^−1^ indicating –N–H stretching. In the ^1^H NMR spectrum of compound **1a**, adoublet at δ 7.78 ppm and amultiplet at δ 7.26–7.11 ppm were assigned to aromatic protons, and a broad singlet at δ 6.12 ppm (br s, 1H) to a –N–H proton. This spectral data allowed determining the structure of compound **1a** as N-Phenyl-1H-benzo[d]imidazol-2-amine, which was further confirmed by its ^13^C NMR spectrum which exposed 11 signals between δ 139.2 and 110.8 ppm, indicates the existence of 11 different carbons in the compound. Further, the formation of compound **1a** was supported by the molecular ion peak at 210.10[M + H]^+^ in its mass spectrum (EI).

**Figure 2:**
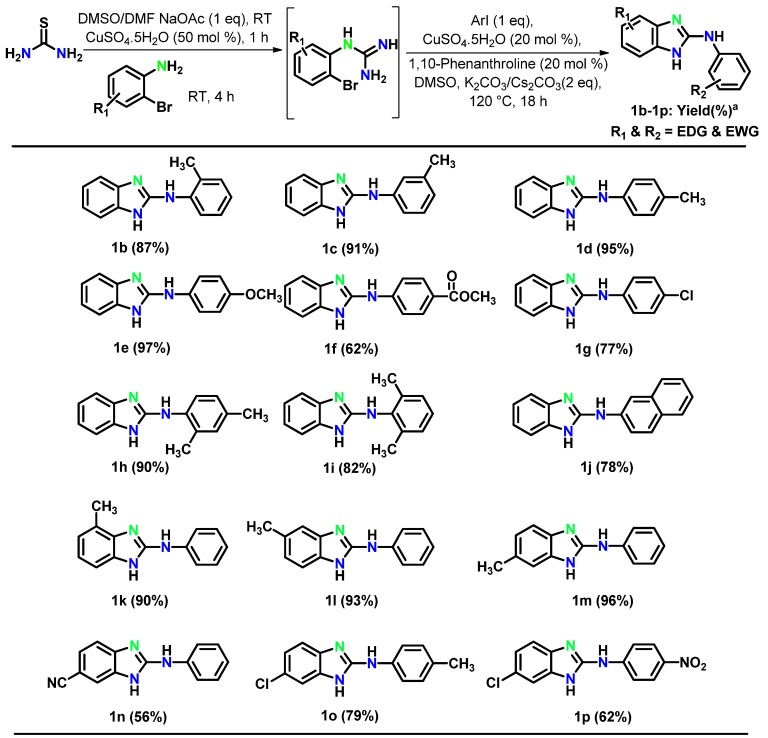
Substrate scope evaluation; Reaction conditions: thiourea (1 mmol); DMSO (2 mL); NaOAc (1 equiv); CuSO_4_·5H_2_O (50 mol%), 1 h, room temperature; 2-bromo aniline/substituted 2-bromo aniline (2 mmol), room temperature, 4 h; Next, ArI (1 mmol); CuSO_4_·5H_2_O (20 mol%); 1,10-Phenanthroline (20 mol%); K_2_CO_3_ (2 mmol); 18 h; 120 °C; ^a^ Isolated yield.

Next, to validate the efficiency of the protocol, we investigated the substrate scope towards the production of 15 more 2-arylaminobenzimidazoles **1b–1p** under optimized conditions, and the results are indicated in Figure 2. *N*-(2-bromophenyl)-guanidine was reacted with various derivatives of aryl iodide having 2-CH_3_, 3-CH_3_, 4-CH_3_, 4-OCH_3_, 4-COOCH_3_, 4-Cl, 2,4-DiCH_3_, 2,6-DiCH_3_ and naphthyl to obtain the final products **1b**–**1j** in 62–97% yields. Similarly, the reaction of 2-bromoaniline holding substituents such as 2-Me, 3-Me, 4-Me, 4-CN readily provided the desired products **1k**–**1n** in 56–96% yields. In addition, several substrates were tested to understand the reactivity of aryl halides. For example, thiourea reacted with 4-chloro-2-bromoaniline to provide guanidine which consecutively underwent a C–N cross-coupling reaction with 4-methyliodobenzene and 4-nitroiodobenzene to afford the target products **1o** and **1p** in 79% and 62% yields respectively. The above mentioned results clearly confirmed that the substrates having electron-donating and electron-withdrawing groups are compatible with this process, affording the substituted 2-aminobenzimidazoles in moderate to excellent yield. All the spectroscopic and analytical data of the synthesized compounds were in full agreement with the anticipated structures.

The reaction of aryl halides was next examined under optimized reaction conditions. Iodobenzene afforded the target product **1a** in 96% yield (Equation (1)). On the other hand, bromobenzene led to the target product **1a** in 12% yield and to the product 2-aminobenzimidazole **B** in 81% yield (Equation (2)). However, no target product could be obtained when using chlorobenzene, which provided 2-aminobenzimidazole **B** in 97% yield (Equation (3)). In addition, we also examined the activity of different haloanilines. It was observed that 2-iodoaniline readily reacted with thiourea to give iodoguanidine **C**, which required a shorter reaction time than bromoguanidine to undergo a domino C–N cross-coupling reaction with iodobenzene, affording the desired product **1a** in 96% yield (Equation (4)). However, 2-chloroaniline gave the intermediate chloroguanidine **D** in complete conversion, but no domin O C–N cross-coupled product was observed with iodobenzene under optimized reaction conditions (Equation (5)).

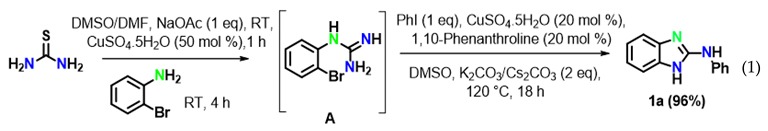


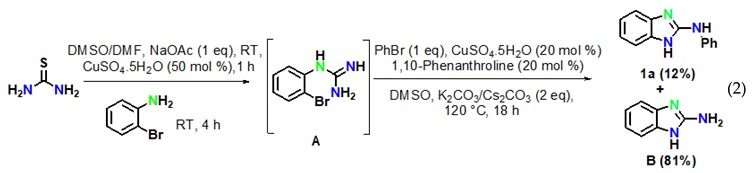


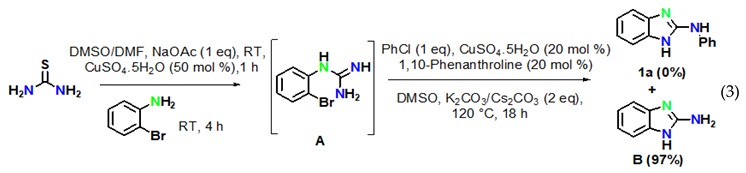


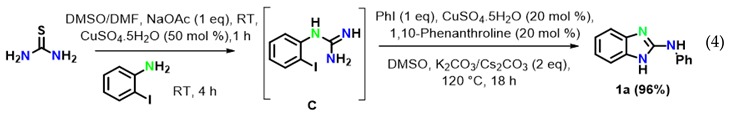


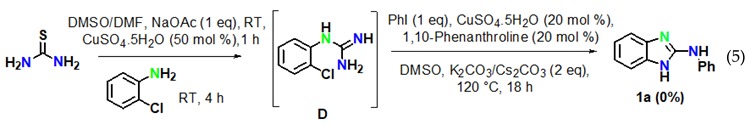



To reveal the mechanism, control experiments were conducted. The reaction was performed in the absence of aryl iodide and resulted inthe intramolecular C–N cyclized product 2-aminobenzimidazole **B** in 97% yield (Equation (6)) under optimized reaction conditions. Furthermore, 2-aminobenzimidazole readily reacted with iodobenzene under optimized conditions to afford the target product **1a** in 96% yield (Equation (7)).

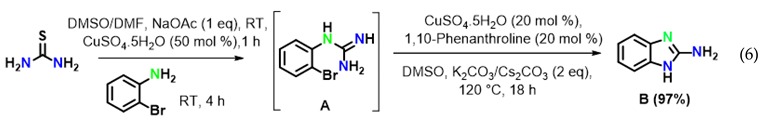


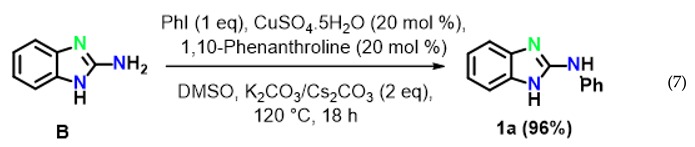



The above results clearly suggested that thiourea treated with 2-bromoaniline to provide N-(2-bromophenyl)-guanidine **A** gave first the intramolecular C–N cyclised product 2-amino benzimidazole **B**, which reacted with iodobenzene to providethe final product **1a** by an intermolecular C–N cross-coupling reaction.

Moreover, the formation of the key intermediate **B** was confirmed based on its spectral data. In fact, in the IR spectrum of compound **B**, thepeaks at 3421(w) and 3396(w) cm^−1^ corresponds to N–H stretching, the peak at 3063(w) cm^−1^ represents –C=C–H stretching, the peaks at 1626(s), 1530(s), 1444(s) cm^−1^ correspond to Ar–C=C stretching; ^1^H-NMR analysis showed a broad signal at δ 5.62(2H) ppm, indicating amino protons and signals between δ7.58 and 7.09 ppm representing aromatic protons. Moreover, in the ^13^C NMR spectrum, seven signals between δ167.0 and 118.2 ppm also confirmed that compound **B** is 1H-benzo[d]imidazol-2-amine. Finally, the structure of **B** was confirmed by the appearanceof a peak at m/z 134.07 [M + H]^+^ in its mass spectrum (EI).

Based on these experimental investigations and the available literature [37,40,41,42,43,44,45,46,47,48,49,50], the mechanism for the formation of the final product 2-phenylaminobenzimidazole is very much similar to the mechanism reported for cobalt catalysis [37] (see supporting information Scheme S1).

## 3. Experimental

### 3.1. Material and Methods

General information: 2-bromoaniline, iodobenzene, CuSO_4_∙5H_2_O (98%), CuI (98%), CuBr (98%), Cu_2_O (97%), CuCl (99%), Cu(OAc)_2_∙H_2_O (98%), sodium acetate, KOH, K_2_CO_3_, and Cs_2_CO_3_ were purchased from Sigma-Aldrich (St. Luis, MO, USA) and were used without further purification. The solvents were purchased and dried according to standard procedures prior to use. The reactions were monitored by using pre-coated Merck60 F_254_ (Merck, Darmstadt, Germany) TLC silica gel plates with 0.25 mm thickness. Column chromatography was performed for purification, utilizing silica gel (60–120 mesh, Merck, Darmsadt, Germany). A Cintex melting point apparatus (Cintex, Mumbai, India) was used to determine the melting points. Infrared (IR) spectra were recorded on a Perkin Elmer Spectrum one FT-IR spectrometer (Perkin Elmer, Waltham, Massachusetts, USA). A Varian 400 MHz spectrometer (Varian, Palo Alto, CA, USA) was utilized to record the ^1^H NMR and ^13^C-NMR spectra in CDCl_3_/DMSO-d_6_. Chemical shifts are presented in δ ppm, employing TMS as internal reference. A Jeol SX-102 spectrometer (JEOL Ltd., Akishima, Tokyo, Japan) was used to record the Mass spectra.

### 3.2. General Procedure for the Synthesis of 2-(N-arylamino)Benzimidazole

To a stirred solution of DMSO (2–3 mL), thiourea (1 mmol, 76 mg) was added slowly, followed by the addition of NaOAc (1 mmol, 82 mg) and a copper source (50 mol%) at room temperature. The whole reaction mixture was stirred for 1 h (until a black-colored solution was obtained) at room temperature. To this, 2-bromoaniline (2 mmol, 344 mg) was added. After completion of the reaction (monitored by TLC), the reaction mixture was transferred into tubes and the mixture was centrifuged for 10 min. A black solid was then recovered from the tubes, and to this a clear solution of iodobenzene (1 mmol, 204 mg), K_2_CO_3_ (2 mmol, 277 mg), Cu(SO)_4_.5H_2_O (20 mol%, 50 mg), and 1,10-phenanthroline (20 mol%, 36 mg) was added slowly in several minutes, and the reaction mixture was then allowed to stir for 18 h at 120 °C. Progress of the reaction was monitored by TLC, using ethyl acetate and hexane (1:4). After completion of the reaction, the reaction mixture was cooled to room temperature. Then, the solution was washed with ethyl acetate (7 mL) and water (3 mL) for 5 times. The organic layer was evaporated, and the crude reaction mixture was purified on asilica gel (60–120 mesh) column by chromatography to obtain the final product 2-(N-arylamino)benzimidazole, which was characterized by NMR (^1^H and ^13^C), IR, and mass spectral data.

*N-Phenyl-1H-benzo[d]imidazol-2-amine* (**1a**): White solid; yield 96%; mp 97–99 °C; ^1^H NMR (400 MHz, CDCl_3_) δ 7.78 (d,*J* = 6.8 Hz, 2H), 7.26–7.11 (m, 7H), 6.12 (br s, 1H); ^13^C NMR (100 MHz, CDCl_3_) δ 139.2, 137.2, 134.2, 132.6, 130.5, 129.9, 129.7, 127.3, 115.0, 114.7, 110.8; FT-IR (KBr) 3294, 3098, 1676, 1618, 1597, 1542, 1478, 1413, 1253, 1076 cm^−1^. *m*/*z* (ESI–MS) 210.10 [M + H]^+^.

*N-O-Tolyl-1H-benzo[d]imidazol-2-amine* (**1b**): White solid; yield 87%; mp 151–152 °C; ^1^H NMR (400 MHz, DMSO) δ 7.35 (d, *J* = 12 Hz, 1H), 7.25–7.19 (m, 2H), 7.00 (d,*J* = 7.4 Hz, 2H), 6.88–6.77 (m, 3H), 2.18 (s, 3H); ^13^C NMR (100 MHz, CDCl_3_) δ 138.2, 137.5, 137.1, 135.7, 133.9, 132.2, 131.6, 130.9, 129.0, 125.3, 123.2, 118.2, 115.2, 20.7; FT-IR (KBr) 3314, 2924, 2859, 3109, 2115, 1619, 1509, 1330, 1250, 1112, 1088, 1025 cm^−1^. *m*/*z* (ESI–MS) 224.11 [M + H]^+^.

*N-m-Tolyl-1H-benzo[d]imidazol-2-amine* (**1c**): White solid; yield 91%; mp 148–149 °C; ^1^H NMR (400 MHz, CDCl_3_) δ 7.55 (s, 1H), 7.43–7.34 (m, 3H), 7.31–7.25 (m, 3H), 6.70 (br s, 1H), 2.36 (s, 3H); ^13^C NMR (100 MHz, CDCl_3_) δ 149.0, 139.4, 137.4, 134.9, 134.7, 133.6, 133.1, 130.5, 124.0, 122.0, 115.5, 113.1, 110.4, 20.6; FT-IR (KBr) 3257, 3191, 2958, 1501, 1495, 1403, 1386, 1341, 1286, 1061 cm^−1^. *m*/*z* (ESI–MS) 224.11 [M + H]^+^.

*N-p-Tolyl-1H-benzo[d]imidazol-2-amine* (**1d**): White solid; yield 95%; mp 145–147 °C; ^1^H NMR (400 MHz, CDCl_3_) δ 7.45–7.32 (m, 6H), 7.12 (d,*J* = 8 Hz, 2H), 5.98 (br s, 1H), 2.29 (s, 3H); ^13^C NMR (100 MHz, CDCl_3_ + DMSO-d6) δ 152.3, 142.2, 135.9, 133.0, 130.8, 129.8, 128.7, 128.5, 123.2, 120.6, 117.8, 20.0; FT-IR (KBr) 3256, 3121, 2963, 1643, 1586, 1514, 1367, 1234, 1136, 1094, 1017 cm^−1^. *m*/*z* (ESI–MS) 224.11 [M + H]^+^.

*N-(4-Methoxyphenyl)-1H-benzo[d]imidazol-2-amine* (**1e**): White solid; yield 97%; mp 153–154 °C; ^1^H NMR (400 MHz, CDCl_3_) δ 7.47–7.42 (m, 3H), 7.30–7.16 (m, 2H), 7.14–7.08 (m, 3H), 7.00 (br s, 1H), 3.87 (s, 3H); ^13^C NMR (100 MHz, CDCl_3_ + DMSO-d6) δ 154.7, 153.1, 142.9, 133.7, 132.6, 129.7, 129.5, 129.3, 126.1, 121.3, 114.9, 55.02; FT-IR (KBr) 3345, 2958, 2857, 1567, 1535, 1506, 1321, 1271, 1235, 1182, 1123, 1074, 1033 cm^−1^. *m*/*z* (ESI–MS) 240.11 [M + H]^+^.

*Methyl 4-(1H-benzo[d]imidazol-2-ylamino)benzoate* (**1f**): White solid; yield 62%; mp 207–209 °C; ^1^H NMR (300 MHz, d6-DMSO, ppm) δ 7.64–7.51 (m, 3H), 6.83–6.61 (m, 5H), 5.83 (br s, 1H), 3.83 (s, 3H);^13^C NMR (75 MHz, d6-DMSO) d = 166.3, 152.7, 145.7, 144.2, 141.3, 132.3, 131.2, 128.1, 119.8, 119.1, 106.6, 106.3, 45.3; FT-IR (KBr) 3234, 3157, 2853, 1658, 1599, 1516, 1428, 1411, 1242, 1197, 1121, 1087, 1065, 1022 cm^−1^. *m*/*z* (ESI-MS) 268.10 [M + H]^+^.

*N-(4-Chlorophenyl)-1H-benzo[d]imidazol-2-amine* (**1g**): White solid; yield 77%; mp 158–159 °C; ^1^H NMR (400 MHz, CDCl_3_) δ 7.77–7.50 (m, 4H), 7.26 (d, *J* = 7.6 Hz, 2H), 7.16 (d, *J*= 8.8 Hz, 2H), 4.66 (br s, 1H); ^13^C NMR (100 MHz, CDCl_3_ + DMSO-d6) δ 151.1, 141.7, 136.9, 132.3, 128.3, 128.1, 127.7, 127.0, 124.7, 120.0, 118.1; FT-IR (KBr) 3076, 2958, 2150, 1637, 1504, 1421, 1374, 1330, 1256, 1207, 1030 cm^−1^. *m*/*z* (ESI–MS) 245.05 [M + H]^+^.

*N-(2,4-Dimethylphenyl)-1H-benzo[d]imidazol-2-amine* (**1h**): White solid; yield 90%; mp 143–144 °C;^1^H NMR (400 MHz, CDCl_3_) δ 7.65 (s, 1H), 7.38–7.33 (m, 3H), 7.27–7.09 (m, 3H), 6.74 (br s, 1H), 2.28 (s, 3H), 2.27 (s, 3H); ^13^C NMR (100 MHz, CDCl_3_) δ 141.3, 138.3, 137.2, 136.9, 132.9, 132.4, 130.6, 127.2, 117.7, 115.1, 111.2, 21.4, 20.4; FT-IR (KBr) 3278, 3201, 2922, 2858, 1607, 1581, 1453, 1410, 1389, 1268, 1155, 1018 cm^−1^. *m*/*z* (ESI–MS) 238.13 [M + H]^+^.

*N-(2,6-Dimethylphenyl)-1H-benzo[d]imidazol-2-amine* (**1i**): White solid; yield 82%; mp 148–149 °C; ^1^H NMR (400 MHz, CDCl_3_) δ 7.22–7.17 (m, 2H), 7.13 (d,*J* = 8.4 Hz, 2H), 7.06–6.97 (m, 3H), 6.35 (br s, 1H), 2.43 (s, 6H), 2.18 (s, 3H); ^13^C NMR (100 MHz, CDCl_3_) δ 142.8, 135.2, 134.4, 134.3, 130.5, 130.1, 129.5, 128.7, 118.4, 115.5, 111.2, 24.5, 20.7; FT-IR (KBr) 3094, 2921, 2867, 2222, 1574, 1486, 1456, 1374, 1241, 1208, 1027 cm^−1^. *m*/*z* (ESI–MS) 238.13 [M + H]^+^.

*N-(Naphthalen-3-yl)-1H-benzo[d]imidazol-2-amine* (**1j**): White solid; yield 78%; mp 168–169 °C; ^1^H NMR (400 MHz, CDCl_3_) δ 8.02–7.63 (m, 5H), 7.51–7.47 (m, 3H), 7.35 (d, *J* = 8 Hz, 1H), 7.26 (d, *J* = 5.2 Hz, 2H), 6.60 (br s, 1H); ^13^C NMR (100 MHz, CDCl_3_ + DMSO-d6) δ 154.6, 142.6, 134.2, 133.8, 133.6, 131.2, 129.4, 129.1, 128.8, 128.3, 127.8, 127.2, 125.8, 125.4, 122.4, 121.1, 120.8. *m*/*z* (ESI–MS) 260.11 [M + H]^+^.

*4-Methyl-N-phenyl-1H-benzo[d]imidazol-2-amine* (**1k**): White solid; yield 90%; mp 151–153 °C;^1^H NMR (400 MHz, CDCl_3_) δ 7.47-7.25 (m, 4H), 7.07 (d, *J* = 7.6 Hz, 2H), 6.94 (d, *J* = 8 Hz, 1H), 6.74 (br s, 1H), 6.54 (s, 1H), 2.35 (s, 3H); ^13^C NMR (100 MHz, CDCl_3_) δ 146.7, 139.5, 135.5, 134.4, 132.8, 129.7, 128.7, 126.4, 124.5, 117.9, 110.0, 21.8; FT-IR (KBr) 3290, 3144, 2984, 2917, 2859, 1604, 1584, 1498, 1449, 1413, 1391, 1289, 1270, 1213, 1153, 1057, 934 cm^−1^. *m*/*z* (ESI–MS) 224.11 [M + H]^+^.

*5-Methyl-N-phenyl-1H-benzo[d]imidazol-2-amine* (**1l**): White solid; yield 93%; mp 151–153 °C; ^1^H NMR (400 MHz, CDCl_3_) δ 7.76–7.49 (m, 4H), 7.25–6.85 (m, 4H), 2.37 (s, 3H); ^13^C NMR (100 MHz, CDCl_3_ + DMSO-d6) δ 151.6, 141.5, 135.7, 134.8, 132.2, 128.3, 128.0, 127.8, 119.9, 118.0, 114.3, 19.1; FT-IR (KBr) 3306, 2920, 2847, 1603, 1515, 1444, 1302, 1236, 1302, 1236, 1191, 1115, 1095, 1020 cm^−1^. *m*/*z* (ESI–MS) 224.11 [M + H]^+^.

*6-Methyl-N-phenyl-1H-benzo[d]imidazol-2-amine* (**1m**): White solid; yield 96%; mp 146–147 °C; ^1^H NMR (400 MHz, CDCl_3_) δ 7.40 (s, 1H), 7.30–7.19 (m, 5H), 7.04–7.01 (m, 2H), 6.81 (br s, 1H), 2.32 (s, 3H); ^13^C NMR (100 MHz, CDCl_3_) δ 138.0, 137.4, 135.3, 134.1, 132.7, 129.6, 127.0, 126.7, 115.3, 110.0, 20.3; FT-IR (KBr) 3285, 3056, 2854, 1603, 1574, 1534, 1497, 1456, 1321, 1234, 1121, 1085 cm^−1^. *m*/*z* (ESI–MS) 224.11 [M + H]^+^.

*2-(Phenylamino)-3H-benzo[d]imidazole-5-carbonitrile* (**1n**): White solid: yield 56%; mp 169–170 °C; ^1^H NMR (400 MHz, CDCl_3_) δ 7.53 (s, 1H), 7.29–7.22 (m, 3H), 7.16 (d, *J* = 6.8 Hz, 2H), 6.97 (d, *J* = 7.2 Hz, 2H), 6.84 (br s, 1H); ^13^C NMR (100 MHz, CDCl_3_) δ 139.2, 137.3, 134.2, 132.6, 130.5, 130.0, 129.7, 127.3, 115.0, 114.8, 111.0, 110.9; FT-IR (KBr) 3253, 3198, 1654, 1588, 1488, 1407, 1317, 1287, 1164, 1019, 927 cm^−1^. *m*/*z* (ESI–MS) 235.09 [M + H]^+^.

*6-Chloro-N-p-tolyl-1H-benzo[d]imidazol-2-amine* (**1o**): White solid: yield 79%; mp 151–152 °C; ^1^H NMR (400 MHz, CDCl_3_) δ 7.77 (s, 1H), 7.50 (t, *J* = 9.2 Hz, 2H), 7.26 (d, *J* = 7.6 Hz, 2H), 7.16 (d, *J* = 8.8 Hz, 2H), 4.66 (br s, 1H), 2.40 (s, 3H); ^13^C NMR (100 MHz, CDCl_3_ + DMSO-d6) δ 151.1, 141.7, 136.9, 132.3, 128.3, 128.1, 127.7, 127.0, 124.7, 120.0, 118.1, 19.2; FT-IR (KBr) 3076, 2958, 2150, 1637, 1504, 1421, 1374, 1330, 1256, 1207, 1030 cm^−1^. *m*/*z* (ESI–MS) 259.12 [M + H]^+^.

*6-Chloro-N-(4-nitrophenyl)-1H-benzo[d]imidazol-2-amine*(**1p**): White solid: yield 62%; mp 178–180 °C; ^1^H NMR (400 MHz, CDCl_3_) δ 7.74 (d, *J* = 2.0 Hz, 1H), 7.47–7.37 (m, 3H), 7.10 (d, *J* = 9.2 Hz, 1H), 6.94 (br s, 1H), 6.81 (dd, *J* = 7.2, 2.4 Hz, 2H); ^13^C NMR (100 MHz, CDCl_3_ + DMSO-d6) δ 153.9, 152.1, 135.8, 131.9, 131.2, 130.1, 129.9, 127.9, 121.8, 119.2, 112.7; FT-IR (KBr) 3275, 3085, 2834, 1607, 1579, 1486, 1302, 1233, 1179, 1085, 1036 cm^−1^. *m*/*z* (ESI–MS) 290.04 [M + H]^+^.

*1H-benzo[d]imidazol-2-amine*(**B**): White solid; mp 129–130 °C; ^1^H NMR (400 MHz, CDCl_3_) δ 7.58–7.56 (td, *J* = 8.0, 0.4 Hz, 1H), 7.53–7.50 (dd, *J* = 8.0, 0.8 Hz, 1H), 7.31–7.27 (m, 1H), 7.13–7.09 (d, *J* = 7.6, 1.2 Hz, 1H), 5.62 (br s, 2H); ^13^C NMR (100 MHz, CDCl_3_) δ 167.0, 151.9, 131.0, 125.5, 121.4, 120.6, 118.2; FT-IR (KBr) 3421, 3396, 3063, 1626, 1530, 1444, 1309, 1105 cm^−1^. *m*/*z* (ESI–MS) 134.07 [M + H]^+^.

## 4. Conclusions

In conclusion, we have developed a methodology for the synthesis of substituted 2-aminophenyl benzimidazoles from thiourea through a multistep reaction. This methodology involves consecutive a desulphurization/nucleophilic substitution and domino intra and inter molecular C–N cross-coupling reaction. Control experiments were performed to understand themechanism. Many researchers have reported reactions for the generationof benzimidazoles; however, the simplicity, environmental acceptability, and cost effectiveness of copper makes this method more practical. Although the single yields look moderate, considering that the reaction consists of multiple processes, the yields are in fact good to high.

## Figures and Tables

**Table 1:** Optimization of the reaction for the synthesis of 2-aminophenyl benzoimidazole ^a^.

Entry	Copper Salt	Base	Temp °C	Ligand	Conversion ^e^ 1a	Yield (%) ^g^
1	CuI	K_2_CO_3_	RT	L4	n.d. ^f^	-
2	CuI	K_2_CO_3_	50	L4	n.d. ^f^	-
3	CuI	K_2_CO_3_	80	L4	20	15
4	CuI	K_2_CO_3_	100	L4	55	52
5	CuI	K_2_CO_3_	120	L4	100	96
6	CuI	KOH	120	L4	71	66
7	CuI	Cs_2_CO_3_^b^	120	L4	100	96
8	CuBr	K_2_CO_3_	120	L4	100	94
9	CuCl	K_2_CO_3_	120	L4	100	96
10	CuSO_4_·5H_2_O	K_2_CO_3_	120	L4	100	95
11	Cu(OAc)_2_·H_2_O	K_2_CO_3_	120	L4	100	96
12	CuSO_4_·5H_2_O	K_2_CO_3_	120	-	10	6
13	CuSO_4_·5H_2_O	K_2_CO_3_	120	L1	30	24
14	CuSO_4_·5H_2_O	K_2_CO_3_	120	L2	50	56
15	CuSO_4_·5H_2_O	K_2_CO_3_	120	L3	15	11
16	CuSO_4_·5H_2_O	K_2_CO_3_	120	L5	55	48
17	CuSO_4_·5H_2_O ^c^	K_2_CO_3_	120	L4	40	36
18	CuSO_4_·5H_2_O	K_2_CO_3_^d^	120	L4	55	52
19	-	K_2_CO_3_	120	L4	n.d. ^f^	-

^a^ Reaction conditions: Step-1: Thiourea (1 mmol); DMSO/DMF (2 mL); NaOAc (1 equiv); copper salt (50 mol%), 1 h, room temperature; 2-bromo aniline (2 mmol), room temperature, 4 h; Step-2: iodo benzene (1 mmol); copper salt (20 mol%); ligand (20 mol%); base (2 mmol) 18 h,temp; ^b^ Cs_2_CO_3_ (2.0 equiv); ^c^ copper salt (10 mol.%); ^d^ K_2_CO_3_ (1.0 equiv); ^e^ conversion based on diagnostic peaks integration in ^1^HNMR of crude reaction mixture; ^f^ n.d. = not detected; ^g^isolated yield.

## Supplementary Materials

The following are available online. Scheme S1. Proposed mechanism.
