# Supplementary material for: Copper-Promoted One-Pot Approach: Synthesis of Benzimidazoles
Source: Molecules. 2020 Apr 14;25(8):1788. doi: 10.3390/molecules25081788 (PMC7221555; doi:10.3390/molecules25081788)
Supplement: Supplementary file 1 [file molecules-25-01788-s001.pdf]

molecules

Supporting Information

Copper Promoted One Pot Approach: Synthesis of Benzimidazoles

S N Murthy Boddapati[1,2], Ramana Tamminana[3], Ravi Kumar Gollapudi[1], Sharmila Nurbasha[1], Mohamed E. Assal[4], Osamah Alduhaish[*,4], Mohammed Rafiq H. Siddiqui[4], Hari Babu Bollikolla[*,1] and Syed Farooq Adil[*,4]

[1]Department of Chemistry, Acharya Nagarjuna University, Guntur, Andhar Pradesh, India-522510.
[2]Department ofChemistry, Sir C. R. Reddy College, PG Courses, Eluru, Andhra Pradesh, India-534002.
[3]Department ofChemistry, GITAM Deemed to be University, Bengaluru Campus, Karnataka, India-562163.
[4]Department of Chemistry, College of Science, King Saud University, P.O. 2455, Riyadh 11451, Kingdom of Saudi Arabia.
*Corresponding authors E-mail: dr.b.haribabu@gmail.com (B. HariBabu); sfadil@ksu.edu.sa (Syed Farooq Adil);oalduhaish@ksu.edu.sa (OsamahAlduhaish)

Mechanism

Based on the experimental evidences and literature reports [1, 2-12] the proposed mechanism for the formation of final product 2-aminophenylbenzimidazole **1a** is as follows.

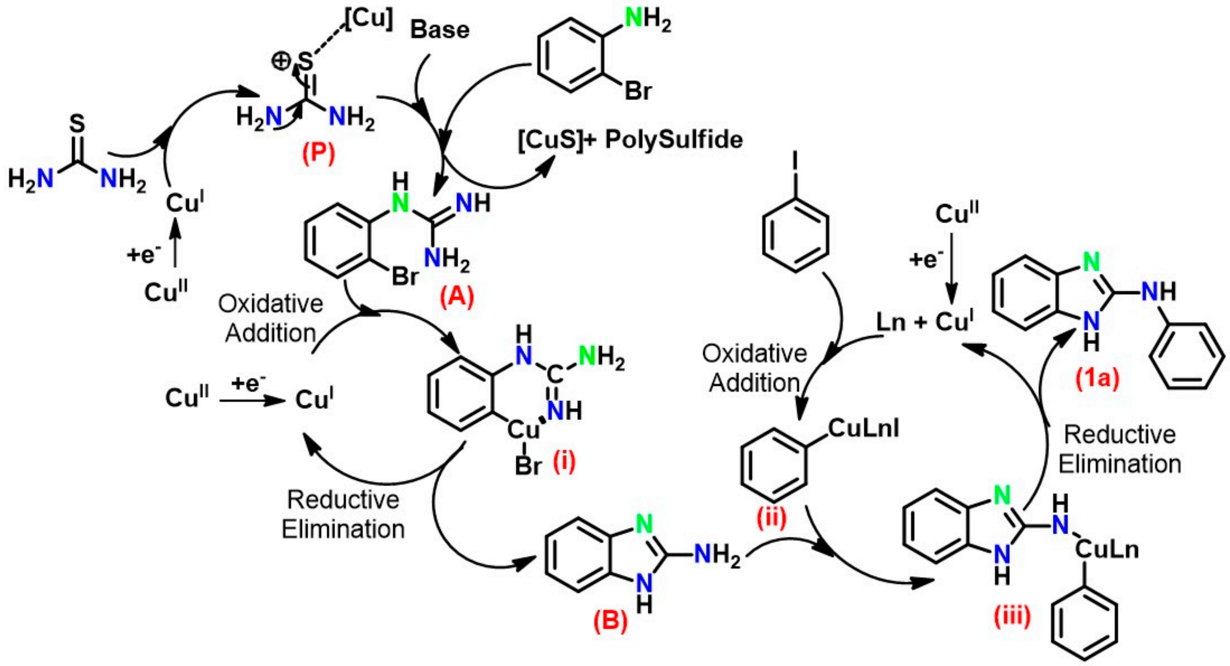

Scheme S1. Proposed mechanism [1]

Initially, the reduction of copper(II) salt with thiourealead to the *in situ* generation of copper(I) species [5], which can coordinate with thiourea to afford intermediate **P**. Next, the intermediate **P** on deprotonation with base followed by reaction with 2-bromoaniline may provide N-(2-bromophenyl)-guanidine **A** along with by-products CuS and polysulphide [2] *via* desulphurization/substitution reactions[3,4]. Oxidative addition of **A** with copper (I) species (it could be generated from copper (II) species [5] can afford intermediate ***(i)***, that may lead to the

formation of intramolecular C-N cyclised product 2- aminobenzimidazole **B**[6,7] using base. On the other hand, oxidative addition of iodobenzene with copper (I) species lead to the formation of intermediate ***(ii)***, which can undergo an intermolecular C-N cross-coupling reaction[8-12] with intermediate **B** to afford the intermediate ***(iii)***that can complete the catalytic cycle by reductive elimination of final product 2-aminophenylbenzimidazole **1a**.

References

1. Kondraganti, L.; Manabolu, S.b.; Dittakavi, R. Synthesis of Benzimidazoles via Domino Intra and Intermolecular C-N Cross-Coupling Reaction. *ChemistrySelect* **2018**, *3*, 11744-11748.
2. Ramana, T.; Punniyamurthy, T. Preparation of 2-Azido-1-Substituted-1 H-Benzo [d] imidazoles Using a Copper-Promoted Three-Component Reaction and Their Further Conversion into 2-Amino and 2-Triazolyl Derivatives. *Chemistry–A European Journal* **2012**, *18*, 13279-13283.
3. Guin, S.; Rout, S.K.; Gogoi, A.; Nandi, S.; Ghara, K.K.; Patel, B.K. Desulfurization strategy in the construction of azoles possessing additional nitrogen, oxygen or sulfur using a copper (I) catalyst. *Advanced Synthesis & Catalysis* **2012**, *354*, 2757-2770.
4. Yella, R.; Khatun, N.; Rout, S.K.; Patel, B.K. Tandem regioselective synthesis of tetrazoles and related heterocycles using iodine. *Organic & biomolecular chemistry* **2011**, *9*, 3235-3245.
5. For the reduction of copper (II) salts to copper (I) species using thiourea, see:Bowmaker, G.A.; Hanna, J.V.; Pakawatchai, C.; Skelton, B.W.; Thanyasirikul, Y.; White, A.H. Crystal structures and vibrational spectroscopy of copper (I) thiourea complexes. *Inorg. Chem.* **2009**, *48*, 350-368.
6. Wang, X.; Kuang, C.; Yang, Q. Copper-Catalyzed Synthesis of 4-Aryl-1H-1, 2, 3-triazoles from 1, 1-Dibromoalkenes and Sodium Azide. *Eur. J. Org. Chem.* **2012**, *2012*, 424-428.
7. Chiba, S.; Zhang, L.; Ang, G.Y.; Hui, B.W.-Q. Generation of iminyl copper species from α-azido carbonyl compounds and their catalytic C− C bond cleavage under an oxygen atmosphere. *Org. Lett.* **2010**, *12*, 2052-2055.
8. Tan, B.Y.-H.; Teo, Y.-C. Efficient cobalt-catalyzed C–N cross-coupling reaction between benzamide and aryl iodide in water. *Organic & biomolecular chemistry* **2014**, *12*, 7478-7481.
9. Ma, D.; Lu, X.; Shi, L.; Zhang, H.; Jiang, Y.; Liu, X. Domino Condensation/S-Arylation/Heterocyclization Reactions: Copper-Catalyzed Three-Component Synthesis of 2-N-Substituted Benzothiazoles. *Angew. Chem. Int. Ed.* **2011**, *50*, 1118-1121.
10. Cahiez, G.; Moyeux, A. Cobalt-catalyzed cross-coupling reactions. *Chem. Rev.* **2010**, *110*, 1435-1462.
11. Boddapati, S.N.M.; Polam, N.; Mutchu, B.R.; Bollikolla, H.B. The synthesis of arylcyanamides: a copper catalyzed consecutive desulfurization and C-N cross coupling strategy.*New J. Chem.***2018**, *42*, 918-922.
12. Boddapati, S.N.M.; Saketi,J.M.R.; Mutchu, B.R.; Bollikolla, H.B.; Adil, S.F.; Khan, M. Copper promoted desulfurization and C-N cross coupling reactions: Simple approach to the synthesis of substituted 2-aminobenzoxazoles and 2,5-disubstituted tetrazole amines. *Arabian Journal of Chemistry,***2020**, *13*, 4477-4494.
